# Role of TLR4/NADPH oxidase/ROS-activated p38 MAPK in VCAM-1 expression induced by lipopolysaccharide in human renal mesangial cells

**DOI:** 10.1186/1478-811X-10-33

**Published:** 2012-11-15

**Authors:** I-Ta Lee, Ruey-Horng Shih, Chih-Chung Lin, Jung-Tsan Chen, Chuen-Mao Yang

**Affiliations:** 1 Department of Anesthetics, Chang Gung Memorial Hospital at Linkou and College of Medicine, Chang Gung University, Kwei-San, Tao-Yuan, Taiwan; 2 Department of Physiology and Pharmacology and Health Aging Research Center, College of Medicine, Chang Gung University, 259 Wen-Hwa 1st Road, Kwei-San, Tao-Yuan, Taiwan

**Keywords:** Lipopolysaccharide, Vascular cell adhesion molecule-1, Toll-like receptors, Reactive oxygen species, NADPH oxidase

## Abstract

**Background:**

In bacteria-induced glomerulonephritis, Toll-like receptor 4 (TLR4) activation by lipopolysaccharide (LPS, a key component of the outer membranes of Gram-negative bacteria) can increase oxidative stress and the expression of vascular cell adhesion molecule-1 (VCAM-1), which recruits leukocytes to the glomerular mesangium. However, the mechanisms underlying VCAM-1 expression induced by LPS are still unclear in human renal mesangial cells (HRMCs).

**Results:**

We demonstrated that LPS induced VCAM-1 mRNA and protein levels associated with an increase in the promoter activity of VCAM-1, determined by Western blot, RT-PCR, and promoter assay. LPS-induced responses were inhibited by transfection with siRNAs of TLR4, myeloid differentiation factor 88 (MyD88), Nox2, Nox4, p47^phox^, c-Src, p38 MAPK, activating transcription factor 2 (ATF2), and p300 or pretreatment with the inhibitors of reactive oxygen species (ROS, edaravone), NADPH oxidase [apocynin (APO) or diphenyleneiodonium chloride (DPI)], c-Src (PP1), p38 MAPK (SB202190), and p300 (GR343). LPS induced NADPH oxidase activation, ROS production, and p47^phox^ translocation from the cytosol to the membrane, which were reduced by PP1 or c-Src siRNA. We observed that LPS induced TLR4, MyD88, c-Src, and p47^phox^ complex formation determined by co-immunoprecipitation and Western blot. We further demonstrated that LPS stimulated ATF2 and p300 phosphorylation and complex formation via a c-Src/NADPH oxidase/ROS/p38 MAPK pathway. Up-regulation of VCAM-1 led to enhancing monocyte adhesion to HRMCs challenged with LPS, which was inhibited by siRNAs of c-Src, p47^phox^, p38 MAPK, ATF2, and p300 or pretreatment with an anti-VCAM-1 neutralizing antibody.

**Conclusions:**

In HRMCs, LPS-induced VCAM-1 expression was, at least in part, mediated through a TLR4/MyD88/ c-Src/NADPH oxidase/ROS/p38 MAPK-dependent p300 and ATF2 pathway associated with recruitment of monocyte adhesion to kidney. Blockade of these pathways may reduce monocyte adhesion via VCAM-1 suppression and attenuation of the inflammatory responses in renal diseases.

## Background

Mesangial cells (MCs) response to various pathological stimuli associated with the main events of glomerular inflammation, including leukocyte infiltration, cell proliferation, and fibrosis, which were predominantly mediated through induction of adhesion molecules [1,2]. In bacteria-induced glomerulonephritis, lipopolysaccharide (LPS, a key component of the outer membranes of Gram-negative bacteria) stimulated VCAM-1 induction in the murine glomerular mesangium [3]. It has been also reported that Toll-like receptor 4 (TLR4) activation by LPS increased the expression of adhesion molecules, such as VCAM-1 which recruits leucocytes to the kidney [4,5].

Reactive oxygen species (ROS) are known to play a prominent role in the pathogenesis of various renal disorders, such as nephropathy [6], renal ischemia [7], and renal fibrosis [8]. Nicotinamide adenine dinucleotide phosphate (NADPH) oxidase is an important enzymatic source for the production of ROS under various pathologic conditions [9]. NADPH oxidase-derived ROS have been shown to induce monocyte chemoattractant protein-1 expression in MCs leading to nephropathy [10]. Activated NADPH oxidase is a multimeric protein complex, including p47^phox^ cytosolic subunits. It has been shown that the phosphorylation of p47^phox^ results in its membrane translocation and activation of NADPH oxidase [11]. It has been reported that ROS generation is necessary for VCAM-1 induction in IL-1β-treated human tracheal smooth muscle cells [12]. The role of ROS in mediating VCAM-1 expression induced by LPS remains to be clarified in human renal mesangial cells (HRMCs).

Src family kinases have been shown to mediate NADPH oxidase activation and ROS generation in lung endothelial cells [13]. c-Src has also been shown to stimulate the phosphorylation of p47^phox^ and therefore increased NADPH oxidase-derived ROS in VCAM-1 expression in IL-1β-treated human tracheal smooth muscle cells [12]. However, the mechanisms underlying NADPH oxidase activation and ROS production regulated by p47^phox^ translocation mediated through c-Src in LPS-induced VCAM-1 expression are also unclear in HRMCs. On the other hand, it has also been shown that ROS stimulate p38 MAPK phosphorylation in opossum kidney cells [14]. However, the role of p38 MAPK in NADPH oxidase-derived ROS-dependent VCAM-1 expression induced by LPS is still unclear in HRMCs.

The promoter region of VCAM-1 possesses a series of functional element, including activator protein-1 (AP-1) binding sites that are essential for induction of VCAM-1 associated with inflammatory responses [15]. It has been established that various stimuli, such as bacterial infections have been shown to induce AP-1 activity [16]. AP-1 is a dimeric protein, consisting of dimers composed of members of either ATF, Jun, or Fos families of proteins [17]. However, the role of ATF2 in LPS-induced VCAM-1 expression is still unknown in HRMCs.

In addressing these questions, experiments were undertaken to investigate the mechanisms underlying LPS-induced VCAM-1 expression mediated through NADPH oxidase activation/ROS generation in HRMCs. These findings suggest that in HRMCs, LPS-induced VCAM-1 expression was, at least in part, mediated through a TLR4/MyD88/c-Src/NADPH oxidase/ROS/p38 MAPK-dependent p300 and ATF2 pathway relevant to recruitment of monocyte adhesion to kidney. These results provide new insights into the mechanisms of LPS action on HRMCs to regulate the expression of VCAM-1 and thus exaggerates the inflammatory responses.

## Results

### LPS induces VCAM-1 expression via a TLR4/MyD88-dependent pathway

**Figure 1:**
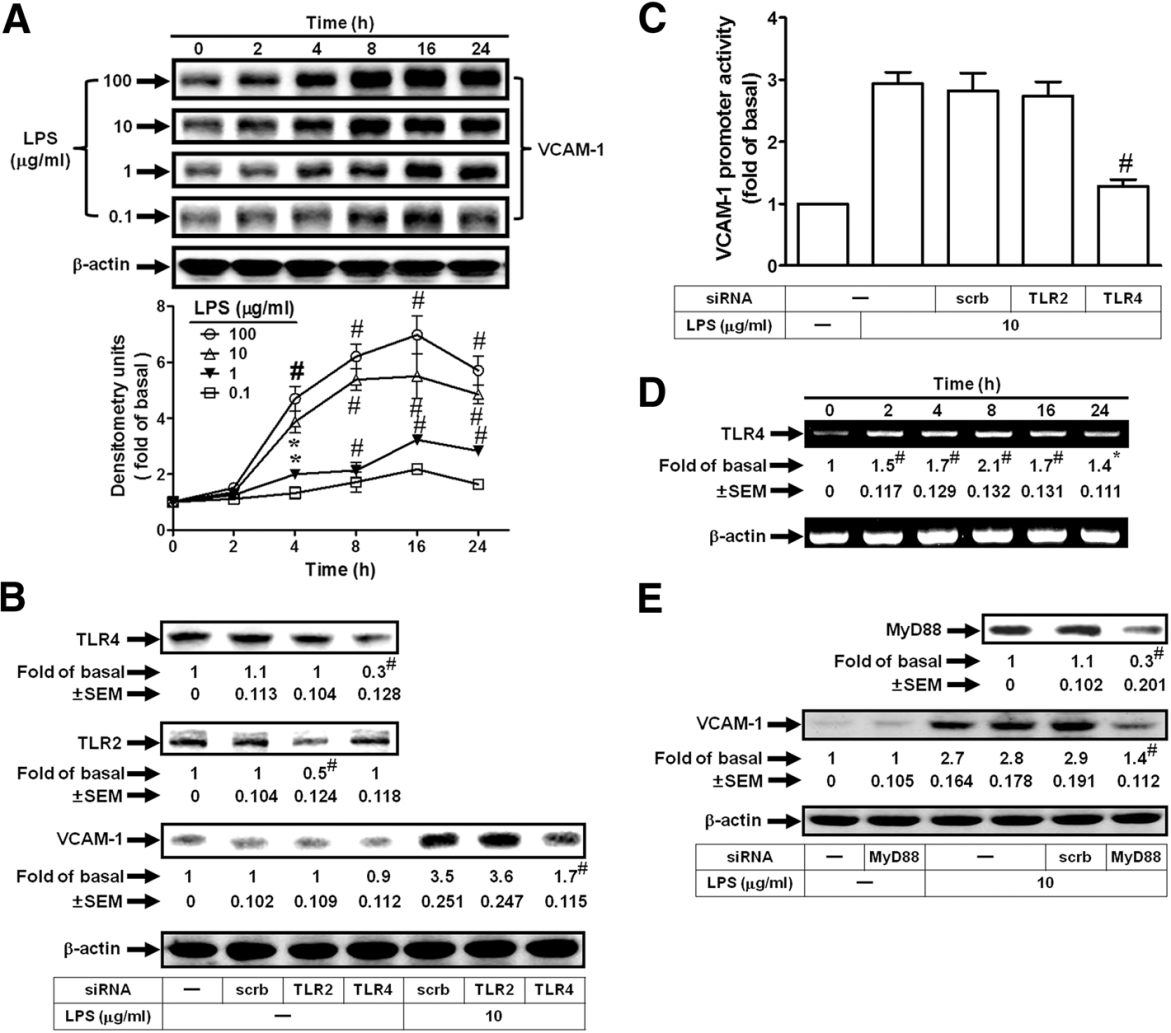
**LPS induces VCAM-1 expression via TLR4/MyD88 in HRMCs.** (**A**) Cells were treated with LPS for the indicated time intervals. The protein levels of VCAM-1 were determined by Western blot. (**B**) Cells were transfected with TLR2 siRNA, TLR4 siRNA, or scrambled siRNA, and then incubated with LPS (10 μg/ml) for 8 h. The levels of TLR4, TLR2, and VCAM-1 protein were determined by Western blot. (**C**) Cells were transiently transfected with VCAM-1-luc reporter gene and TLR2 siRNA, TLR4 siRNA, or scrambled siRNA, and then incubated with LPS for 6 h. The VCAM-1 promoter activity was determined in the cell lysates. (**D**) Cells were incubated with LPS (10 μg/ml) for the indicated time intervals. The mRNA expression of TLR4 was determined by RT-PCR. (**E**) Cells were transfected with MyD88 siRNA or scrambled siRNA, and then incubated with LPS (10 μg/ml) for 8 h. The levels of MyD88 and VCAM-1 protein were determined by Western blot. All figures are representative of three independent experiments, performed in duplicate or triplicate. Data are expressed as means ± SEM. *P < 0.05; ^#^*P* < 0.01, as compared with the cells exposed to vehicle alone (**A**, **D**). ^#^*P* < 0.01, as compared with the cells exposed to scrambled siRNA alone [**B** (upper panel), **E** (upper panel)] or LPS + scrambled siRNA [**B** (lower panel), **C**, **E** (lower panel)].

To investigate the effects of LPS on VCAM-1 expression, HRMCs were treated with various concentrations of LPS (100, 10, 1, and 0.1 μg/ml). As shown in Figure 1A, LPS markedly induced VCAM-1 expression in a time- and concentration-dependent manner in HRMCs. TLR4 is an essential signaling receptor for LPS [18]. Indeed, we also demonstrated that LPS-induced VCAM-1 expression was inhibited by transfection with TLR4 siRNA, but not TLR2 siRNA in HRMCs (Figure 1B). In addition, LPS-induced VCAM-1 promoter activity was also reduced by transfection with TLR4 siRNA (Figure 1C). On the other hand, we demonstrated that LPS could directly induce TLR4 mRNA expression in a time-dependent manner in HRMCs (Figure 1D). The TLR4 signaling cascade initiated following LPS binding is enhanced by homodimerization of the receptor and subsequent recruitment of TIR domain-containing adaptor molecules (TIRAP) to the cytoplasmic domain of the receptor [19]. These adaptors include myeloid differentiation factor 88 (MyD88), MyD88 adaptor-like protein (Mal), TIR-containing adaptor inducing IFNβ (TRIF), also known as TIRAP-1 (TICAM-1), and TRIF-related adaptor molecule (TRAM). Activation of TLR4 leads to stimulation of both MyD88-dependent and MyD88-independent pathways [19]. Moreover, in HRMCs, we showed that LPS-induced VCAM-1 expression was inhibited by transfection with MyD88 siRNA (Figure 1E). These results suggested that LPS induced VCAM-1 expression through a TLR4/MyD88-dependent signaling pathway.

### LPS induces NADPH oxidase activation and ROS production in HRMCs

**Figure 2:**
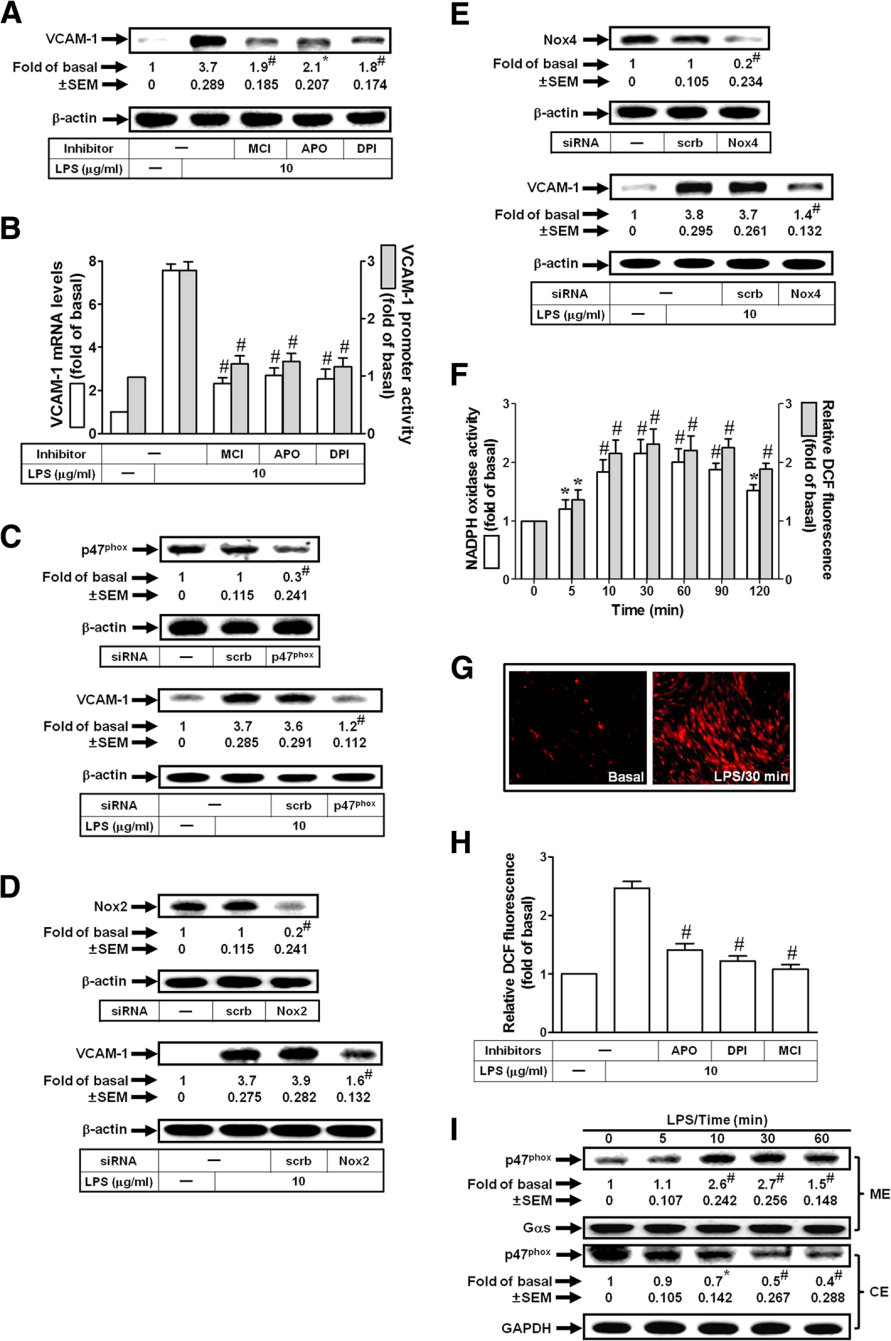
**LPS induces VCAM-1 expression via NADPH oxidase/ROS.** (**A**) Cells were pretreated with MCI-186 (MCI, 100 μM), APO (100 μM), or DPI (10 μM), and then incubated with LPS for 8 h. The levels of VCAM-1 protein were determined. (**B**) Cells were pretreated with MCI-186 (MCI), APO, or DPI, and then incubated with LPS for 4 h or 6 h. The mRNA levels and promoter activity of VCAM-1 were determined. (**C**, **D**, **E**) Cells were transfected with p47^phox^, Nox2, Nox4, or scrambled siRNA, and then incubated with LPS for 8 h. The protein levels of p47^phox^, Nox2, Nox4, and VCAM-1 were determined. (**F**) Cells were stimulated with LPS for the indicated times. NADPH oxidase and ROS generation were measured. (**G**) DHE fluorescence image after 30 min of stimulation with LPS. (**H**) Cells were pretreated with MCI-186 (MCI), APO, or DPI, and then treated with LPS for 30 min. ROS generation was measured. (**I**) Cells were stimulated with LPS for the indicated times. The membrane and cytosolic fractions were prepared and subjected to Western blot using an anti-p47^phox^ antibody. Gsα and GAPDH were used as a marker protein for membrane and cytosolic fractions, respectively. All figures are representative of three independent experiments, performed in duplicate. Data are expressed as means ± SEM. *P < 0.05; ^#^*P* < 0.01, as compared with the cells exposed to LPS alone (**A**, **B**, **H**), scrambled siRNA alone (upper panels of **C**, **D**, **E**), LPS + scrambled siRNA (lower panels of **C**, **D**, **E**), or vehicle alone (**F**, **I**).

NADPH oxidase is an important enzymatic source for the production of ROS under various pathologic conditions [9]. LPS has been shown to activate NADPH oxidase and stimulate ROS generation in human tracheal smooth muscle cells [18]. Here, we investigated whether LPS-induced VCAM-1 expression was mediated through NADPH oxidase/ROS. As shown in Figsure 2A and B, pretreatment with the inhibitor of NADPH oxidase [apocynin (APO) or diphenyleneiodonium chloride (DPI)] or a ROS scavenger (edaravone, MCI-186) markedly inhibited LPS-induced VCAM-1 protein and mRNA expression and promoter activity in HRMCs. Activated NADPH oxidase is a multimeric protein complex consisting of at least three cytosolic subunits of p47^phox^, p67^phox^, and p40^phox^. Phosphorylation of p47^phox^ leads to a conformational change allowing its interaction with p22^phox^[20]. It has been demonstrated that p47^phox^ organizes the translocation of other cytosolic factors, hence its designation as “organizer subunit” [20]. Here, we showed that transfection with p47^phox^ siRNA inhibited LPS-mediated VCAM-1 induction (Figure 2C). Indeed, in cultured HRMCs, Nox2, Nox4, and Nox5 were expressed (data not shown). Moreover, in this study, we also observed that transfection with siRNA of Nox2 or Nox4 markedly reduced LPS-induced VCAM-1 expression in HRMCs (Figures 2D and E). Thus, we suggested that LPS-induced ROS generation was, at least in part, mediated via Nox2 or Nox4 activation in these cells. We further demonstrated that LPS stimulated NADPH oxidase activation and ROS, including H_2_O_2_ and O_2_^·−^ production in HRMCs (Figures 2F and G). Moreover, pretreatment with APO, DPI, or edaravone inhibited LPS-enhanced ROS generation in HRMCs (Figure 2H), suggesting that LPS stimulated ROS production via NADPH oxidase activation. We next investigated the effect of LPS on translocation of p47^phox^ in HRMCs. Cells were treated with 10 μg/ml LPS for the indicated time intervals. The membrane and cytosolic fractions were prepared and subjected to Western blot analysis using an anti-p47^phox^ antibody. As shown in Figure 2I, LPS stimulated a time-dependent increase in translocation of p47^phox^ from the cytosol to the membrane. These data demonstrated that LPS induced ROS generation through a NADPH oxidase-dependent signaling leading to VCAM-1 expression in HRMCs.

### LPS enhances NADPH oxidase activation and ROS generation via c-Src in HRMCs

**Figure 3:**
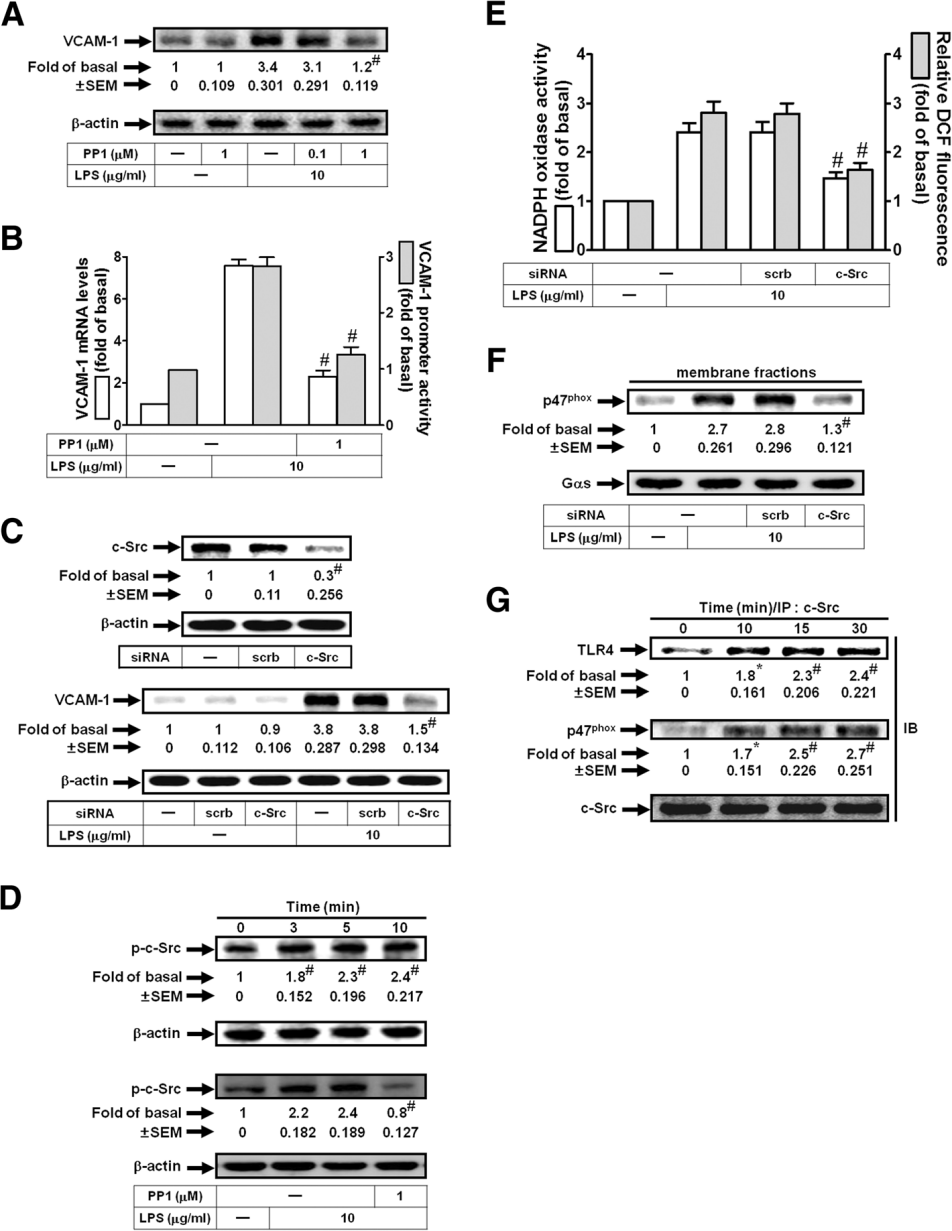
**LPS induces VCAM-1 expression via c-Src.** (**A**) Cells were pretreated with PP1, and then incubated with LPS for 8 h. VCAM-1 protein levels were determined. (**B**) Cells were pretreated with PP1, and then incubated with LPS for 4 h or 6 h. The mRNA levels and promoter activity of VCAM-1 were determined. (**C**) Cells were transfected with c-Src siRNA, and then incubated with LPS for 8 h. The protein levels of c-Src and VCAM-1 were determined. (**D**) Cells were pretreated without or with PP1 (1 μM), and then treated with LPS for the indicated times or 10 min. The expression of phospho-c-Src was determined. (**E**) Cells were transfected with c-Src siRNA, and then stimulated with LPS for 30 min. The NADPH oxidase activity and ROS generation were determined. (**F**) Cells were transfected with c-Src siRNA, and then incubated with LPS for 30 min. The membrane fractions were prepared and subjected to Western blot using an anti-p47^phox^ antibody. (**G**) Cells were treated with LPS for the indicated times. The cell lysates were subjected to immunoprecipitation using an anti-c-Src antibody, and then the immunoprecipitates were analyzed by Western blot using an anti-TLR4, anti-p47^phox^, or anti-c-Src antibody. All figures are representative of three independent experiments, performed in duplicate. Data are expressed as means ± SEM. *P < 0.05; ^#^*P* < 0.01, as compared with the cells exposed to LPS alone [**A**, **B**, **D** (lower panel)], scrambled siRNA alone (**C**, upper panel), LPS + scrambled siRNA [C (lower panel), **E**, **F**], or vehicle alone [**D** (upper panel), **G**].

Recent studies have shown that TLR4 signaling is coupled to c-Src family kinase activation, tyrosine phosphorylation of zonula adherens proteins, and opening of the paracellular pathway in human lung microvascular endothelia [21,22]. We investigated whether c-Src was involved in the induction of VCAM-1 in response to LPS. As shown in Figures 3A and B, pretreatment with the inhibitor of c-Src (PP1) reduced LPS-induced VCAM-1 protein and mRNA expression and promoter activity. In addition, transfection with c-Src siRNA also inhibited LPS-induced VCAM-1 expression (Figure 3C). LPS could stimulate c-Src phosphorylation, which was inhibited by pretreatment with PP1 (Figure 3D). c-Src has been shown to regulate ROS generation in human tracheal smooth muscle cells [9]. Moreover, we also found that LPS-induced p47^phox^ translocation, NADPH oxidase activation, and ROS generation were inhibited by transfection with c-Src siRNA (Figures 3E and F). We further investigated the physical association of TLR4, c-Src, and p47^phox^ in LPS-induced ROS generation and VCAM-1 expression. As shown in Figure 3G, the protein levels of TLR4 and p47^phox^ were time-dependently increased in c-Src-immunoprecipitated complex in LPS-treated HRMCs. Thus, these data suggested that LPS-induced VCAM-1 expression is mediated through c-Src-dependent NADPH oxidase/ROS generation in HRMCs.

### LPS induces VCAM-1 expression via NADPH oxidase/ ROS-dependent p38 MAPK activation in HRMCs

**Figure 4:**
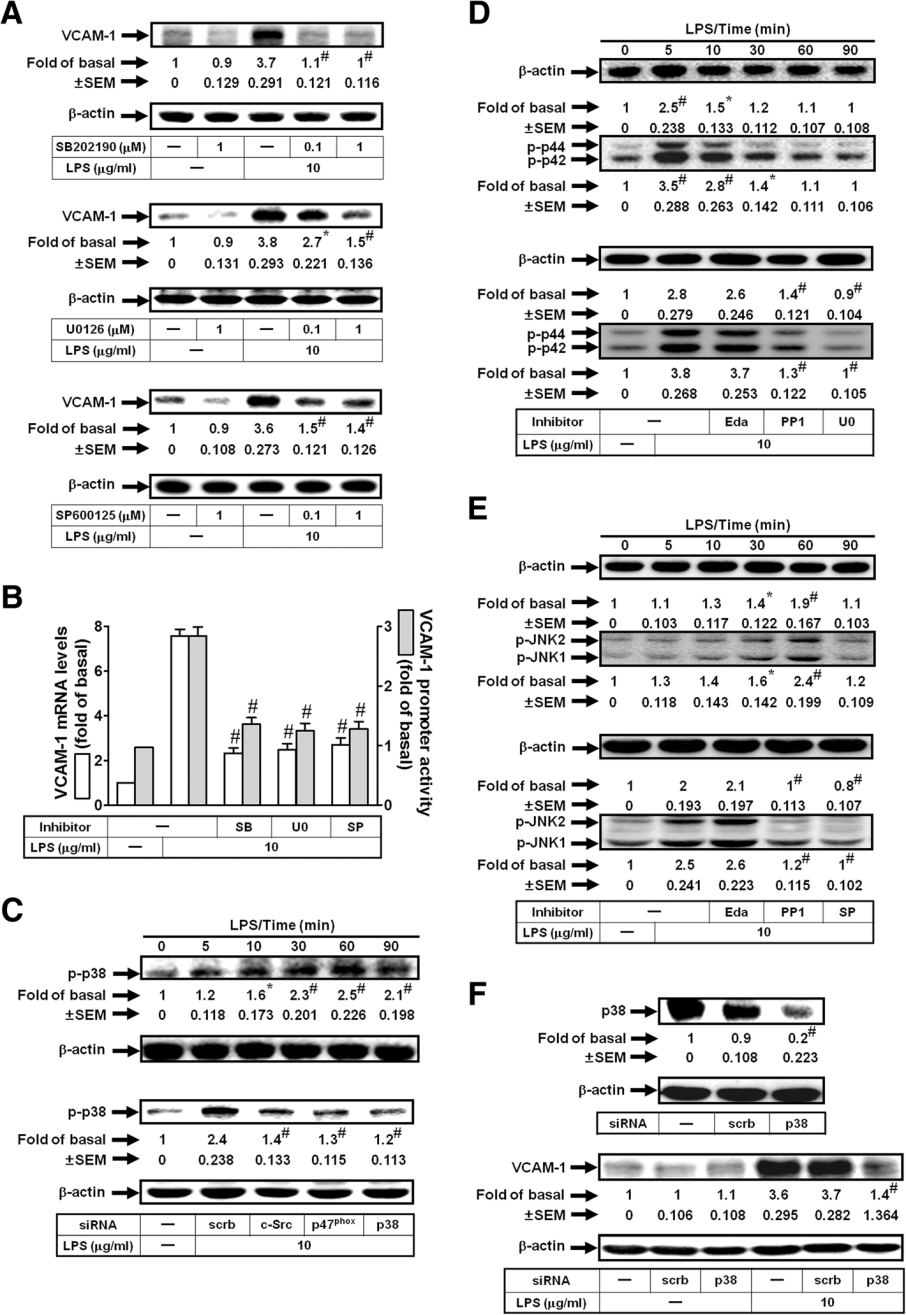
**LPS induces VCAM-1 expression via ROS/p38 MAPK.** (**A**) Cells were pretreated with SB202190, U0126, or SP600125, and then incubated with LPS for 8 h. VCAM-1 protein levels were determined. (**B**) Cells were pretreated with SB202190, U0126, or SP600125, and then incubated with LPS for 4 h or 6 h. mRNA levels and promoter activity of VCAM-1 were determined. (**C**) Cells were treated with LPS for the indicated times or transfected with siRNA of c-Src, p47^phox^, or p38 MAPK, and then treated with LPS for 1 h. Phospho-p38 MAPK expression was determined. (**D**) Cells were treated with LPS for the indicated times or pretreated with edaravone (Eda, 100 μM), PP1 (1 μM), or U0126 (U0, 1 μM), and then treated with LPS for 5 min. Phospho-p42/p44 MAPK expression was determined. (**E**) Cells were treated with LPS for the indicated times or pretreated with edaravone (Eda), PP1, or SP600125 (SP), and then treated with LPS for 1 h. The expression of phospho-JNK1/2 was determined. (**F**) Cells were transfected with p38 MAPK siRNA, and then incubated with LPS for 8 h. Protein levels of p38 MAPK and VCAM-1 were determined. All figures are representative of three independent experiments, performed in duplicate. Data are expressed as means ± SEM. *P < 0.05; ^#^*P* < 0.01, as compared with the cells exposed to LPS alone (**A**, **B**, lower panels of **D** and **E**), vehicle alone (upper panels of **C**, **D**, **E**), scrambled siRNA alone (**F**, upper panel), or LPS + scrambled siRNA (lower panels of **C** and **F**).

MAPKs, including p38 MAPK, JNK1/2, and p42/p44 MAPK have been shown to regulate VCAM-1 induction in various cell types [20,23-25]. Here, we determined whether these three MAPKs were involved in LPS-induced VCAM-1 expression in HRMCs. As shown in Figures 4A and B, pretreatment with the inhibitor of p38 MAPK (SB202190), JNK1/2 (SP600125), or MEK1/2 (U0126) markedly inhibited LPS-induced VCAM-1 protein and mRNA expression and promoter activity in HRMCs. It has been shown that ROS-dependent activation of MAPKs is required for inflammatory responses [26-28]. In HRMCs, LPS-stimulated p38 MAPK phosphorylation was inhibited by transfection with either c-Src siRNA or p47^phox^ siRNA (Figure 4C). However, pretreatment with PP1, but not edaravone inhibited LPS-induced p42/p44 MAPK and JNK1/2 phosphorylation (Figures 4D and E). Finally, the involvement of p38 MAPK in LPS-induced VCAM-1 expression was further confirmed by transfection with p38 MAPK siRNA. As shown in Figure 4F, transfection with p38 siRNA reduced the expression of total p38 MAPK protein and subsequently attenuated VCAM-1 expression induced by LPS. These results indicated that p38 MAPK phosphorylation involved in VCAM-1 induction by LPS was mediated through a c-Src/NADPH oxidase/ROS-dependent cascade in HRMCs.

### LPS induces VCAM-1 expression via p38 MAPK-dependent ATF2 activation

**Figure 5:**
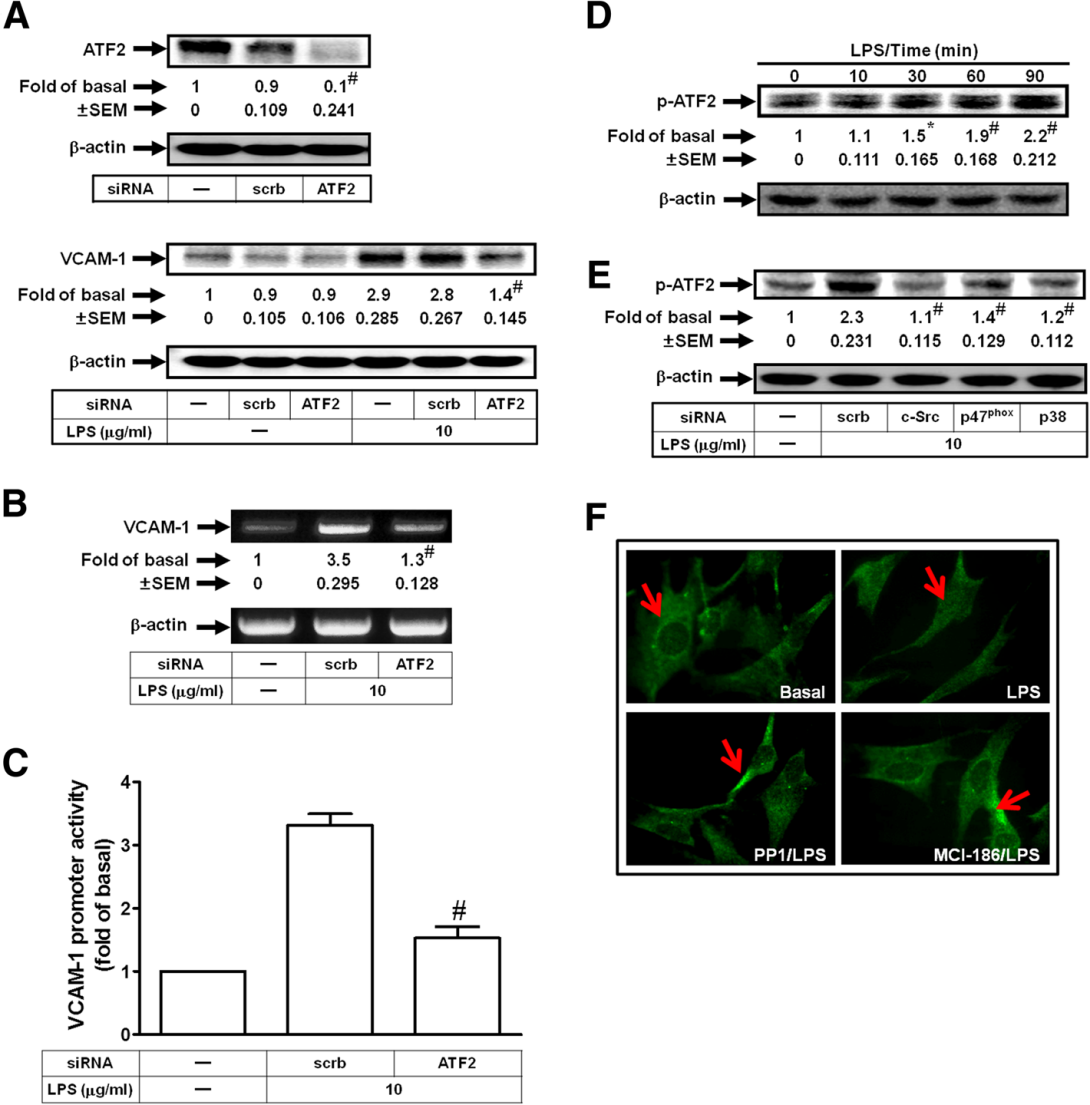
**LPS induces VCAM-1 expression via p38 MAPK/ATF2 in HRMCs.** Cells were transfected with ATF2 siRNA or scrambled siRNA, and then incubated with LPS for (**A**) 8 h or (**B**) 4 h. The levels of VCAM-1 protein (**A**) and mRNA (**B**) expression were determined. (**C**) Cells were transiently transfected with VCAM-1-luc reporter gene, transfected with ATF2 siRNA or scrambled siRNA, and then incubated with LPS for 6 h. The VCAM-1 promoter activity was determined. (**D**) Cells were treated with LPS for the indicated time intervals. The cell lysates were subjected to Western blot using an anti-phospho-ATF2 or anti-β-actin antibody. (**E**) Cells were transfected with siRNA of c-Src, p47^phox^, p38 MAPK, or scrambled, and then treated with LPS for 90 min. The cell lysates were subjected to Western blot using an anti-phospho-ATF2 or anti-β-actin antibody. (**F**) Cells were pretreated with MCI-186 (100 μM) or PP1 (1 μM) for 1 h, and then treated with LPS for 90 min. Cells were fixed, labeled with an anti-ATF2 antibody, and then FITC-conjugated secondary antibody. Individual cells were imaged. All figures are representative of three independent experiments, performed in duplicate or triplicate. Data are expressed as means ± SEM. ^#^*P* < 0.01, as compared with the cells exposed to scrambled siRNA alone (**A**, upper panel) or LPS + scrambled siRNA (**A**, lower panel and **E**). ^#^*P* < 0.01, as compared with the cells exposed to LPS alone (**B**, **C**); *P < 0.05; ^#^*P* < 0.01, as compared with the cells exposed to vehicle alone (**D**).

ATF2 is activated by inflammatory signals transduced by the p38 MAPK pathway [29]. In addition, LPS has also been shown to regulate VCAM-1 expression via an ATF2 signaling [30]. In this study, we investigated whether ATF2 activation was involved in LPS-induced VCAM-1 expression in HRMCs. As shown in Figures 5A, B and C, transfection with ATF2 siRNA inhibited LPS-induced VCAM-1 protein and mRNA expression and promoter activity in HRMCs. On the other hand, we demonstrated that LPS time-dependently stimulated ATF2 phosphorylation, which was inhibited by transfection with siRNA of c-Src, p47^phox^, or p38 MAPK in HRMCs (Figures 5D and E). We found that LPS induced ATF2 translocation from the cytosol to the nucleus, which was inhibited by pretreatment with either PP1 or edaravone (MCI-186) (Figure 5F). These data suggested that ATF2 phosphorylation involved in LPS-induced VCAM-1 expression is mediated through c-Src/NADPH oxidase/ROS/p38 MAPK pathway in HRMCs.

### LPS induces VCAM-1 expression via the formation of an ATF2/p300 complex

**Figure 6:**
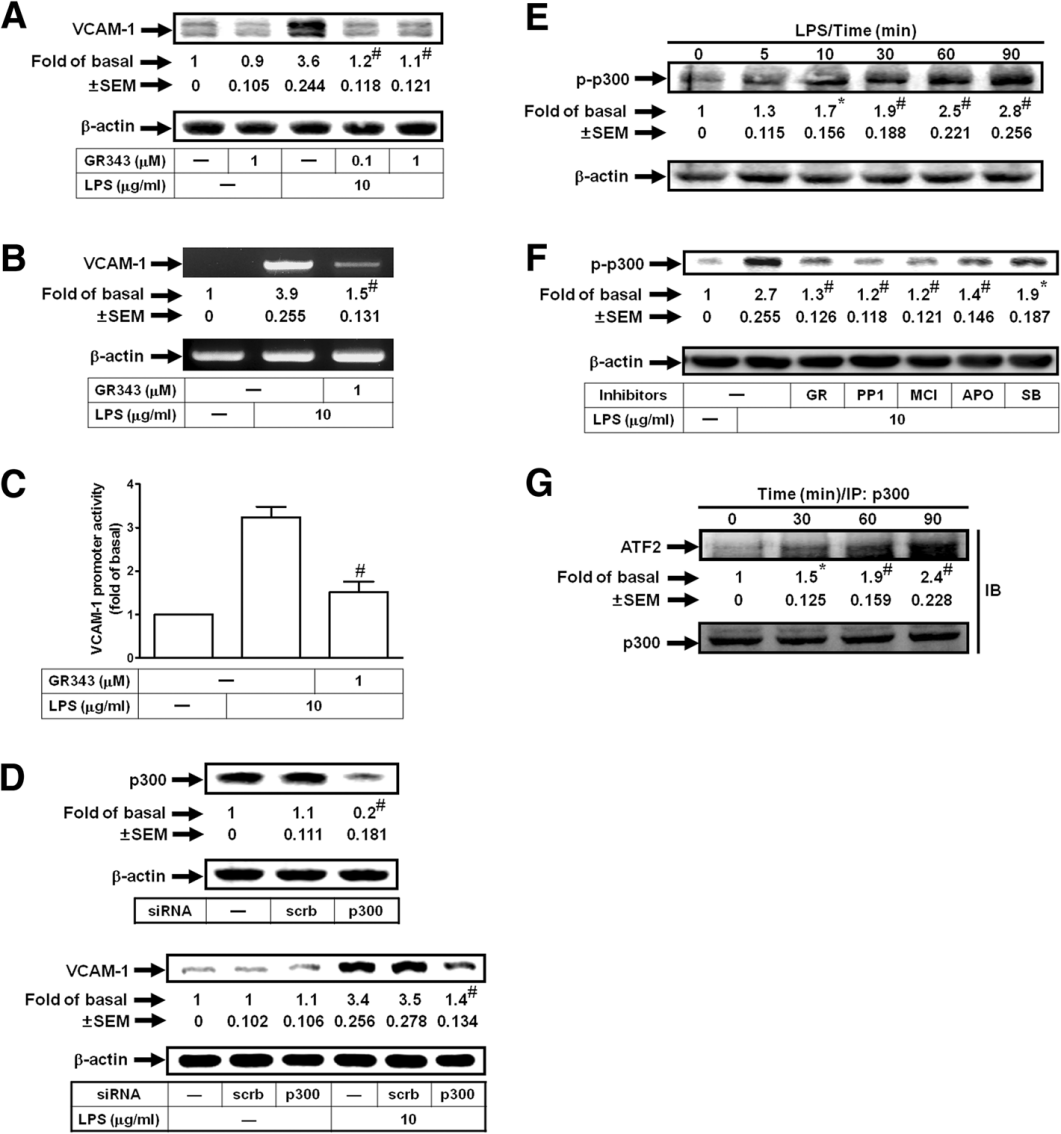
**LPS induces VCAM-1 expression via p300.** Cells were pretreated with GR343, and then incubated with LPS for (**A**) 8 h or (**B**) 4 h. Levels of VCAM-1 protein (**A**) and mRNA (**B**) expression were determined. (**C**) Cells were pretreated with 1 μM GR343, and then incubated with LPS for 6 h. VCAM-1 promoter activity was determined. (**D**) Cells were transfected with p300 siRNA, and then incubated with LPS for 8 h. Protein levels of p300 and VCAM-1 were determined. (**E**) Cells were treated with LPS for the indicated times. The cell lysates were subjected to Western blot using an anti-phospho-p300 antibody. (**F**) Cells were pretreated with GR343 (GR, 1 μM), PP1 (1 μM), MCI-186 (MCI, 100 μM), APO (100 μM), or SB202190 (SB, 1 μM), and then treated with LPS for 90 min. The cell lysates were subjected to Western blot using an anti-phospho-p300 antibody. (**G**) Cells were treated with LPS for the indicated times. The cell lysates were subjected to immunoprecipitation using an anti-p300 antibody, and then the immunoprecipitates were analyzed by Western blot using an anti-ATF2 or anti-p300 antibody. All figures are representative of three independent experiments, performed in duplicate. Data are expressed as means ± SEM. *P < 0.05; ^#^*P* < 0.01, as compared with the cells exposed to LPS alone (**A**, **B**, **C**, **F**), scrambled siRNA alone (**D**, upper panel), LPS + scrambled siRNA (**D**, lower panel), or vehicle alone (**E**, **G**).

p300 has been shown to be involved in VCAM-1 induction [12]. Here, we investigated whether LPS could induce VCAM-1 expression via p300 in HRMCs. As shown in Figures 6A, B and C, pretreatment with the inhibitor of p300 (GR343) significantly reduced LPS-induced VCAM-1 protein and mRNA expression and promoter activity. On the other hand, we also demonstrated that transfection with p300 siRNA down-regulated p300 protein levels and LPS-induced VCAM-1 expression (Figure 6D). LPS also stimulated p300 phosphorylation in a time-dependent manner in HRMCs, which was inhibited by pretreatment with GR343, PP1, edaravone (MCI-186), apocynin, or SB202190 (Figures 6E and F). We further investigated the physical association between p300 and ATF2 in LPS-treated HRMCs. As shown in Figure 6G, cells were stimulated with 10 μg/ml LPS for the indicated time intervals. The cell lysates were subjected to immunoprecipitation using an anti-p300 antibody, and then the immunoprecipitates were analyzed by Western blotting using an anti-p300 or anti-ATF2 antibody. The protein levels of ATF2 were time-dependently increased in p300-immunoprecipitated complex. These results suggested that LPS triggered the interaction between p300 and ATF2 leading to VCAM-1 expression in HRMCs.

### Induction of VCAM-1 enhances adhesion of THP-1 cells to HRMCs challenged with LPS

**Figure 7:**
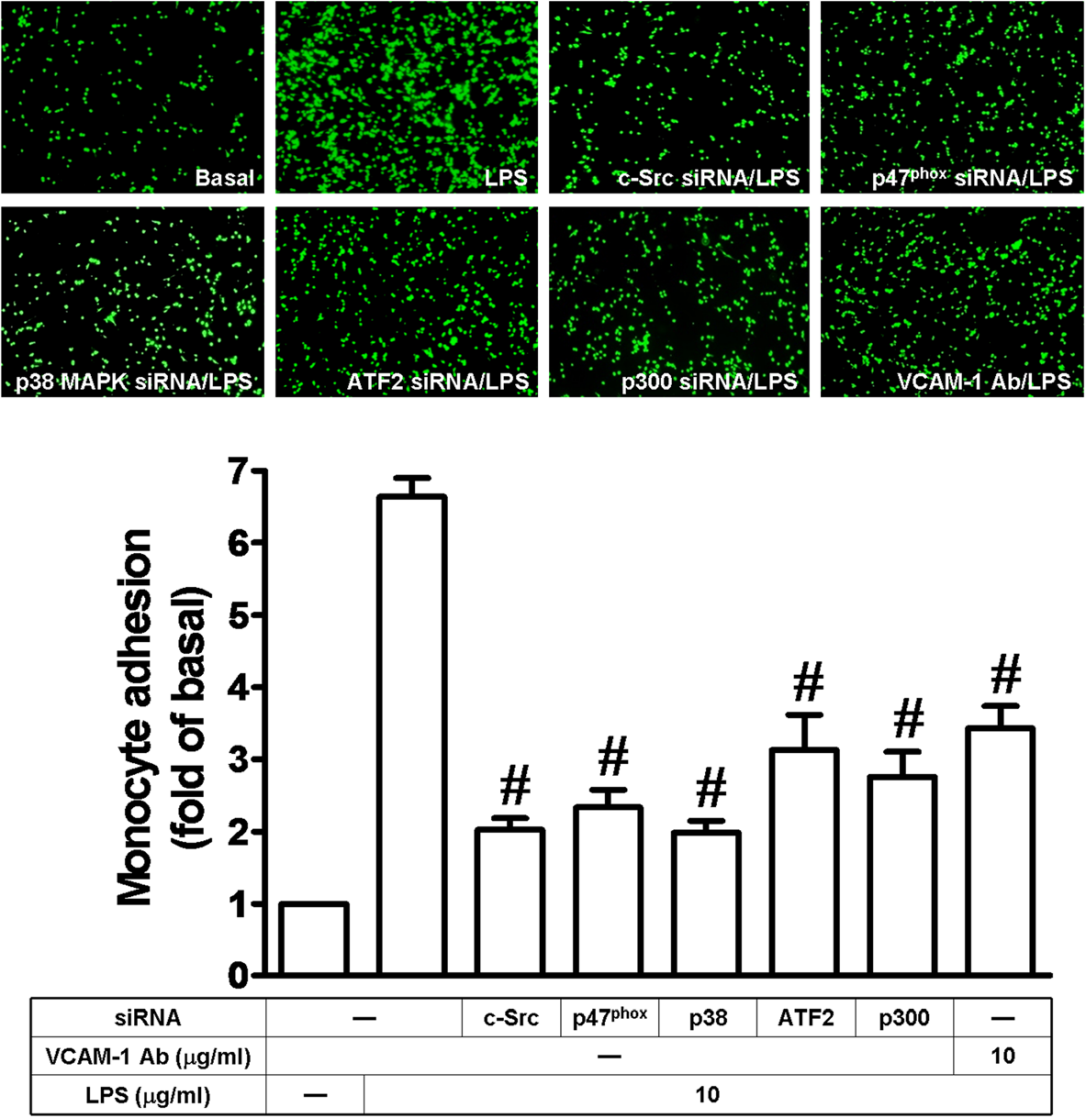
**LPS enhances the adhesion of THP-1 cells to HRMCs.** Cells were transfected with siRNA of c-Src, p47^phox^, p38 MAPK, ATF2, p300 or scrambled, or pretreated with anti-VCAM-1 Ab, and then incubated with LPS for 16 h. The THP-1 cells adherence was measured. All figures are representative of three independent experiments, performed in duplicate. Data are expressed as means ± SEM. ^#^*P* < 0.01, as compared with the cells exposed to LPS alone.

We investigated the roles of c-Src, p47^phox^, p38 MAPK, ATF2, and p300 in the adhesion of THP-1 cells to HRMCs challenged with LPS. As shown in Figure 7, transfection with siRNAs of c-Src, p47^phox^, p38 MAPK, ATF2, and p300 or preincubation with an anti-VCAM-1 neutralizing antibody markedly inhibited the adhesion of THP-1 cells to HRMCs treated with LPS.

## Discussion

**Figure 8:**
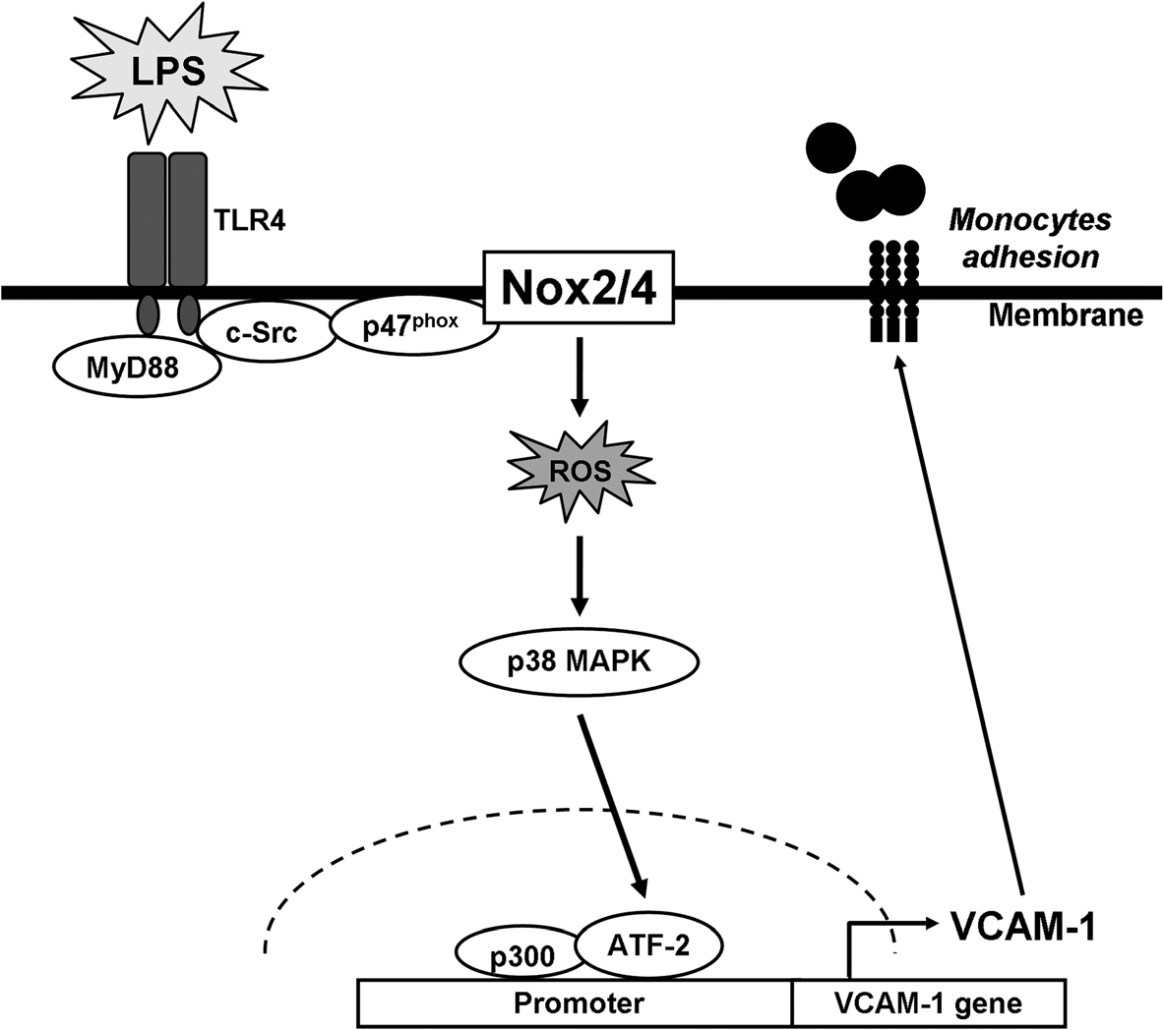
**Schematic representation of the signaling pathways involved in the LPS-induced inflammatory responses.** LPS induced ROS production through TLR4/MyD88/c-Src/NADPH oxidase, in turn initiates the activation of p38 MAPK and ATF2. Activated ATF2 was recruited to the promoter region of VCAM-1 leading to an increase of VCAM-1 promoter activity and the expression of VCAM-1. Up-regulation of VCAM-1 enhances the adhesion of THP-1 ells to HRMCs challenged with LPS.

LPS has been shown to stimulate TNF-α production and ICAM-1 and VCAM-1 expression leading to renal inflammatory diseases [3]. LPS-induced VCAM-1 expression has been shown to be mediated through MAPKs, AP-1, and NF-κB in various cells types [31,32]. It has been reported that NADPH oxidase/ROS generation is necessary for VCAM-1 induction [12]. Thus, these signaling components may regulate VCAM-1 induction in response to LPS in HRMCs. However, the detail mechanisms underlying LPS-induced VCAM-1 expression in HRMCs remain largely unknown. In this study, our results demonstrated that LPS-induced VCAM-1 expression and the adhesion of THP-1 cells to HRMCs were mediated through the p38 MAPK-dependent p300/ATF2 pathway, which was transactivated by a TLR4/MyD88-dependent c-Src/NADPH oxidase/ROS cascade in these cells (Figure 8).

TLRs are type I transmembrane receptors that expressed on the cell membrane induced by LPS [33]. More than 10 human TLRs have been identified [19]. Moreover, we demonstrated that LPS induced VCAM-1 expression via TLR4 in HRMCs. LPS further directly induced TLR4 gene expression, suggesting that LPS could stimulate kidney inflammation via TLR4 induction. MyD88 is a cytosolic adapter molecule connecting TLRs and IL-1Rs to the interleukin-1 receptor-associated kinase (IRAK) complex. The MyD88- and IRAK-4-dependent TIR (Toll/IL-1R) pathways lead to the production of pro-inflammatory cytokines. All human TLRs other than TLR3 use both MyD88 and IRAK-4 to transduce signals [19]. We showed that LPS induced VCAM-1 expression via a TLR4/MyD88-dependent signaling in HRMCs. In the future, we will further investigate whether IRAK-1, IRAK-4, or TRAF6 involves in VCAM-1 induction.

Oxidative stress, induced by systemic and intrarenal generation of ROS can directly exert renal parenchymal damage and may intensify renal microvascular and functional dysregulation, with a feedforward loop of hypoxia and ROS generation. Moreover, ROS have been shown to cause cellular damage or tissue injury, and then mediate the pathogenesis of various renal disorders, such as renal ischemia or nephropathy [7,10]. The NADPH oxidase family members are proteins that transfer electrons across biological membranes [9]. In general, the electron acceptor is oxygen and the product of the electron transfer reaction is a superoxide [20]. Therefore, the biological function of NADPH oxidase enzymes might be attributable to the production of ROS [20]. Here, we showed that LPS-induced VCAM-1 expression was inhibited by pretreatment with the inhibitor of NADPH oxidase or a ROS scavenger, suggesting that NADPH oxidase/ROS are involved in LPS-induced inflammatory responses. Activated NADPH oxidase is a multimeric protein complex consisting of at least three cytosolic subunits of p47^phox^, p67^phox^, and p40^phox^. The p47^phox^ regulatory subunit plays a critical role in acute activation of NADPH oxidase; phosphorylation of p47^phox^ is thought to relieve the inhibitory intracellular interactions and permit the binding of p47^phox^ to p22^phox^, thereby increasing oxidase activation [9,20]. Moreover, we found that transfection with p47^phox^ siRNA markedly reduced LPS-induced VCAM-1 expression. In addition, LPS also increased the production of H_2_O_2_ and superoxide and the activation of NADPH oxidase in HRMCs. LPS directly stimulated p47^phox^ translocation from the cytosol to the membrane. These results indicated that ROS play a key role in LPS-induced VCAM-1 expression. In renal mesangial cells, Nox1-5 are expressed [34,35]. However, in cultured HRMCs, we only observed that Nox2, Nox4, and Nox5 were expressed (data not shown). Here, we showed that transfection with siRNA of Nox2 or Nox4 markedly reduced LPS-induced VCAM-1 expression in HRMCs. Thus, we suggested that LPS-induced ROS generation was, at least in part, mediated via Nox2 or Nox4 activation in these cells. In the future, we will investigate the detail mechanisms of LPS-regulated Nox2, Nox4, and Nox5 activation and ROS generation in cultured HRMCs.

Src family kinases are signaling enzymes that have long been recognized to regulate critical cellular processes, such as proliferation, survival, migration, and metastasis [36]. c-Src has been shown to regulate VCAM-1 expression in various cell types [12,37]. In addition, NADPH oxidase/ROS have been shown to be mediated through c-Src activation [9,12]. We also established that LPS-induced VCAM-1 expression, p47^phox^ translocation, NADPH oxidase activity, and ROS generation was reduced by c-Src inhibition, suggesting that LPS induced VCAM-1 expression via c-Src/NADPH oxidase/ROS in HRMCs. Nox4 was shown to interact with TLR4 and to be required for LPS-induced ROS production [38,39]. It has been shown that Nox2 is required for TLR4-mediated ROS generation [40]. Here, we found that LPS stimulated the formation of TLR4/c-Src/p47^phox^ complex. Therefore, we suggested that LPS could stimulate the protein-protein interactions among TLR4, c-Src, and Nox2 or Nox4, and then increase the generation of ROS. Although the detail protein-protein interactions among TLR4, c-Src, and p47^phox^ are not known, our results are the first time to show a novel role of TLR4/MyD88/c-Src/p47^phox^ complex formation in LPS-induced NADPH oxidase activation and ROS production in HRMCs. In the future, we will further determine which domains of TLR4, MyD88, c-Src, and p47^phox^ are involved in protein-protein interactions caused by LPS.

The MAPKs regulate diverse cellular programs by relaying extracellular signals to intracellular responses. In mammals, there are more than a dozen MAPK enzymes that coordinately regulate cell proliferation, differentiation, motility, and survival. The best known are the conventional MAPKs, which include the extracellular signal-regulated kinases 1 and 2 (ERK1/2), c-Jun amino-terminal kinases 1 to 3 (JNK1 to −3), p38 (α, β, γ, and δ), and ERK5 families [41]. MAPKs also have been shown to regulate VCAM-1 induction [23,25]. Moreover, this is confirmed by our observation that LPS-induced VCAM-1 expression was reduced by inhibition of p38 MAPK, JNK1/2, or p42/p44 MAPK. ROS have been shown to stimulate p38 MAPK activation [42]. In this study, we demonstrated that LPS-stimulated p38 MAPK, but not p42/p44 MAPK or JNK1/2 activation was mediated through NADPH oxidase/ROS in HRMCs. Thus, we suggested that p38 MAPK mainly plays a key role in LPS-induced NADPH oxidase/ROS-dependent VCAM-1 expression. AP-1 proteins are implicated in the regulation of various cellular processes including proliferation and survival, differentiation, growth, apoptosis, cell migration, and transformation [43]. AP-1 refers to a mixture of dimers formed between members of the Jun, Fos, and ATF families. Moreover, p38 MAPK has been shown to mediate ATF2 phosphorylation [44]. Here, we showed that LPS markedly induced ATF2 activation, which was reduced by p38 MAPK inhibition. Thus, we demonstrated that LPS induced VCAM-1 expression via ROS/p38 MAPK/ATF2 in HRMCs.

The transcriptional coactivator p300 is a ubiquitous nuclear phosphoprotein and transcriptional cofactor with intrinsic acetyltransferase activity. p300 controls the expression of numerous genes in a cell-type and signal-specific manner, and plays a pivotal role in cellular proliferation, apoptosis, and embryogenesis [45]. By catalyzing acetylation of histones and transcription factors, p300 plays a significant role in epigenetic regulation. Recent evidence suggests that abnormal p300 function is associated with deregulated target gene expression, and is implicated in inflammation [45,46]. This is confirmed by our observation that LPS-induced VCAM-1 expression was reduced by inhibition of p300. Moreover, LPS directly stimulated p300 phosphorylation and the formation of ATF2/p300 complex via c-Src/ROS/p38 MAPK. Taken together, we demonstrated that LPS could trigger renal inflammation via p300-dependent VCAM-1 induction.

## Conclusions

In summary, as depicted in Figure 8, our results showed that in HRMCs, LPS induced ROS production through TLR4/MyD88/c-Src/Nox2 or Nox4, in turn initiates the activation of p38 MAPK and ATF2. Activated ATF2 was recruited to the promoter region of VCAM-1 leading to an increase of VCAM-1 promoter activity and the expression of VCAM-1. These results provide new insights into the mechanisms of LPS action on HRMCs to regulate the expression of VCAM-1 and thus exaggerated the inflammation responses.

## Methods

### Materials

Anti-VCAM-1, anti-TLR2, anti-TLR4, anti-MyD88, anti-Nox2, anti-Nox4, anti-p47^phox^, anti-Gsα, anti-c-Src, anti-β-actin, anti-p38 MAPK, anti-ATF2, and anti-p300 antibodies were from Santa Cruz (Santa Cruz, CA). Anti-GAPDH antibody was from Biogenesis (Boumemouth, UK). Anti-phospho-p38 MAPK, anti-phospho-p42/p44 MAPK, anti-phospho-JNK1/2, anti-phospho-c-Src, anti-phospho-ATF2, and anti-phospho-p300 antibodies were from Cell Signaling (Danver, MA). Diphenyleneiodonium chloride (DPI), SP600125, U0126, SB202190, GR343, and PP1 were from Biomol (Plymouth Meeting, PA). 5-(and-6)-chloromethyl-2’,7’-dichlorodihydrofluorescein diacetate, acetyl ester (CM-H_2_DCFDA), 2’,7’-bis-(2-carboxyethyl)-5-(and-6)-carboxyfluorescein, acetoxymethyl ester (BCECF/AM), and dihydroethidium were from Molecular Probes (Eugene, OR). Edaravone (MCI-186) was from Tocris Bioscience (Ellisville, MO). Apocynin (APO) was purchased from ChromaDex (Santa Ana, CA). LPS, enzymes, and other chemicals were from Sigma (St. Louis, MO).

### Cell culture

Human renal mesangial cells (HRMCs) were from ScienCell Research Laboratories (San Diego, CA). Cells were cultured in DMEM/F12 supplemented with 10% FBS and antibiotics (100 U/ml penicillin G, 100 μg/ml streptomycin, and 250 ng/ml fungizone) at 37°C in a humidified 5% CO_2_ atmosphere. Experiments were performed with cells from passages 4 to 8.

### Measurement of intracellular ROS accumulation

The intracellular H_2_O_2_ levels were determined by measuring fluorescence of DCFH-DA, and the O_2_^·−^ levels were determined by measuring the fluorescence of DHE. The fluorescence intensities of DCF and DHE staining were detected at 495/529 and 518/605 nm, respectively, using a fluorescence microscope (Zeiss, Axiovert 200 M). In addition, HRMCs were washed with warm HBSS and incubated in HBSS containing 10 μM DCFH-DA or DHE at 37°C for 30 min. and then replaced with a fresh medium. HRMCs were incubated with various concentrations of LPS for the indicated time intervals. Cells were washed twice with PBS and detached with trypsin/EDTA, and the fluorescence intensity of the cells was analyzed using a FACScan flow cytometer (BD Biosciences, San Jose, CA) at 518 nm excitation and 605 nm emission for DHE and at 495-nm excitation and 529 nm emission for DCF.

### Determination of NADPH oxidase activity by chemiluminescence assay

After incubation with LPS, cells were gently scraped and centrifuged at 400 × *g* for 10 min at 4°C. The cell pellet was resuspended with 35 μl/per well of ice-cold RPMI-1640 medium, and the cell suspension was kept on ice. To a final 200 μl volume of pre-warmed (37°C) RPMI-1640 medium containing either NADPH (1 μM) or lucigenin (20 μM), 5 μl of cell suspension (0.2 × 10^5^ cells) was added to initiate the reaction followed by immediate measurement of chemiluminescence in an Appliskan luminometer (Thermo®) in an out-of-coincidence mode. Appropriate blanks and controls were established, and chemiluminescence was recorded. Neither NADPH nor NADH enhanced the background chemiluminescence of lucigenin alone (30–40 counts per min). Chemiluminescence was continuously measured for 12 min, and the activity of NADPH oxidase was expressed as counts per million cells.

### Western blot analysis

Growth-arrested cells were incubated with LPS at 37°C for the indicated time intervals. The cells were washed, scraped, collected, and centrifuged at 45000 × *g* at 4°C for 1 h to yield the whole cell extract, as previously described [9]. Samples were denatured, subjected to SDS-PAGE using a 12% running gel, and transferred to nitrocellulose membrane. Membranes were incubated with an anti-VCAM-1 antibody for 24 h, and then incubated with an anti-mouse horseradish peroxidase antibody for 1 h. The immunoreactive bands were detected by ECL reagents.

### RT-PCR analysis

Total RNA was isolated with Trizol according to the protocol of the manufacturer. The cDNA obtained from 0.5 μg total RNA was used as a template for PCR amplification as previously described [9]. The primers used were as follows: 5’-TGACGGGGTCACCCACACTGTGCCCATCTA-3’ (sense) and 5’-CTAGAAGCATTTGCGGTGGACGATG-3’ (anti-sense) for β-actin; 5’-GGAACCTTGCAGCTTACAGTGACAGAGCTCCC-3’ (sense) 5’-CAAGTCTACATATCACCCAAG-3’ (anti-sense) for VCAM-1; and ;5’-TGGATACGTTTCCTTATAAG-3’ (sense) and 5’-GAAATGGAGGCACCCCTTC-3’ (anti-sense) for TLR4.

### Real-time RT-PCR analysis

Total RNA was extracted using TRIzol reagent. mRNA was reverse-transcribed into cDNA and analyzed by real-time RT-PCR. Real-time PCR was performed using SYBR Green PCR reagents (Applied Biosystems, Branchburg, NJ) and primers specific for VCAM-1 and GAPDH mRNAs. The levels of VCAM-1 expression were determined by normalizing to GAPDH expression.

### Transient transfection with siRNAs

The small interfering RNA (siRNA) duplexes corresponding to human Nox2, Nox4, TLR2, TLR4, MyD88, p47^phox^, c-Src, p38 MAPK, ATF2, and p300 and scrambled siRNA were from Invitrogen (Carlsbad, CA). Transient transfection of siRNAs was carried out using Metafectene transfection reagent from Biontex Lab (GmbH, Planegg/Martinsried, Germany). siRNA (100 nM) was formulated with Metafectene transfection reagent according to the manufacturer's instruction.

### Isolation of cell fractions

Cells were harvested, sonicated for 5 s at output 1.5 with a sonicator (Misonix Inc., Farmingdale, NY), and centrifuged at 8000 rpm for 15 min at 4°C. The pellet was collected as the nuclear fraction. The supernatant was centrifuged at 14000 rpm at 4°C for 60 min to yield the pellet (membrane fraction) and the supernatant (cytosolic fraction).

### Measurement of VCAM-1 luciferase activity

For construction of the VCAM-1-luc plasmid, human VCAM-1 promoter, a region spanning −1716 to −119 bp (kindly provided by Dr. W.C. Aird, Department of Molecular Medicine, Beth Israel Deaconess Medical Center, Boston, MA) was cloned into pGL3-basic vector (Promega, Madison, WI). VCAM-1-luc activity was determined using a luciferase assay system, as previously described [25] (Promega, Madison, WI).

### Adhesion assay

HRMCs were grown to confluence in 6-well plates with coverslips, incubated with LPS for 16 h, and then adhesion assays were performed. Briefly, THP-1 cells (human acute monocytic leukemia cell line) were labeled with a fluorescent dye, 10 μM BCECF/AM, at 37°C for 1 h in RPMI-1640 medium (Gibco BRL, Grand Island, NY) and subsequently washed with PBS followed by centrifugation. Confluent HRMCs in 6-well plates were incubated with THP-1 cells (2 × 10^6^ cells/ml) at 37°C for 1 h. Non-adherent THP-1 cells were removed and plates were gently washed twice with PBS. The numbers of adherent THP-1 cells were determined by counting four fields per 200X high-power field well using a fluorescence microscope (Zeiss, Axiovert 200 M). Experiments were performed in triplicate and repeated at least three times.

### Co-immunoprecipitation assay

Cell lysates containing 1 mg of protein were incubated with 2 μg of an anti-c-Src or anti-p300 antibody at 4°C for 24 h, and then 10 μl of 50% protein A-agarose beads was added and mixed at 4°C for 24 h. The immunoprecipitates were collected and washed thrice with a lysis buffer without Triton X-100. 5X Laemmli buffer was added and subjected to electrophoresis on SDS-PAGE, and then blotted using an anti-TLR4, anti-p47^phox^, anti-c-Src, anti-p300, or anti-ATF2 antibody.

### Analysis of data

Data were estimated using a GraphPad Prism Program (GraphPad, San Diego, CA). Quantitative data were expressed as the means ± SEM and analyzed by one-way ANOVA followed with Tukey’s post-hoc test. *P* < 0.05 was considered significant.

## Abbreviations

AP-1: Activator protein-1; HRMCs: Human renal mesangial cells; LPS: Lipopolysaccharide; MCs: Mesangial cells; NADPH: Nicotinamide adenine dinucleotide phosphate; ROS: Reactive oxygen species; TLRs: Toll-like receptors; VCAM-1: Vascular cell adhesion molecule-1.

## Competing interests

The authors declare that they have no competing interests.

## Author’s contributions

ITL and RHS designed and performed experiments, acquisition and analysis of data, and drafted the manuscript. CCL and JTC helped to perform experiments and prepare the manuscript. CMY has conceived of the study, participated in its design and coordination, has been involved in drafting the manuscript and revising it critically for important intellectual content and has given final approval of the version to be published. All authors have read and approved the final version of this manuscript.
